# Deep Learning-Based Identification of Pathogenicity Genes in *Phytophthora infestans* Using Time-Series Transcriptomics

**DOI:** 10.3390/plants15020178

**Published:** 2026-01-06

**Authors:** Yinfei Dai, Shihao Lu, Jie Fan, Mengjiao Qiao, Yuheng Zhu, Enshuang Zhao, Hao Zhang

**Affiliations:** 1 College of Computer Science and Technology, Jilin University, Changchun 130012, Chinayhzhu23@mails.jlu.edu.cn (Y.Z.); zhaoes22@mails.jlu.edu.cn (E.Z.); 2 College of Computer Science and Technology, Changchun University, Changchun 130022, Chinaqiaomengjiaolemon@outlook.com (M.Q.)

**Keywords:** potato late blight, *Phytophthora infestans*, time-series transcriptomic, LSTM–Transformer, deep learning

## Abstract

Potato (*Solanum tuberosum* L.) is the world’s fourth most important food crop, and despite China producing nearly one quarter of the global yield, its potato production is severely constrained by late blight. Identifying genes associated with pathogenicity is essential for breeding resistant cultivars and strengthening plant protection strategies. Traditional approaches based on differential expression and statistical modeling often fail to capture temporal dynamics or provide interpretable insights. Here, we introduce an LSTM–Transformer hybrid model designed for data-driven discovery of pathogenicity-related genes from gene expression time-series. The analysis was performed on a time-series expression dataset comprising 32,917 genes across 18 samples (three infection time points × six biological replicates per condition). In this study, we identified 200 high-confidence pathogenicity-related genes from potato infection time-series data. These genes are enriched in 15 biologically meaningful pathways, including plant immunity signaling, reactive oxygen species regulation, secondary metabolic processes, and stress-responsive transcriptional programs. Several newly uncovered candidates participate in defense hormone pathways and cell wall modification, suggesting previously unrecognized roles in late blight susceptibility and resistance. By revealing functional groups and regulatory signatures that characterize pathogenicity, this work provides valuable molecular targets for developing late blight-resistant cultivars. The framework integrates a biologically informed temporal–attention architecture, a gene time-series-specific data partitioning strategy, and an interpretable deep analysis module. A final methodological contribution is the use of a temporal attention-based analytical framework that enables reliable gene prioritization from time-series expression data.

## 1. Introduction

Potato (*Solanum tuberosum* L.) is a common dietary source of carbohydrates worldwide, and China is the largest potato producer globally [1,2,3]. Although tubers infected with late blight—caused by *Phytophthora infestans*—do not directly threaten human health, this pathogen severely damages potato plants and can cause yield losses of up to 80% in severe cases, posing a serious risk to food security [4,5]. The pathogenic mechanism of late blight involves complex molecular interactions between the host and the pathogen; thus, identifying key pathogenicity-associated genes is essential for elucidating infection mechanisms and developing disease-resistance strategies [6,7,8].

Traditional approaches to gene identification mainly rely on differential expression analysis (e.g., RNA-seq using DESeq2) and statistical machine learning models such as support vector machines (SVMs) and Random Forests [9,10]. However, these methods exhibit notable limitations: 1. they fail to capture the dynamic temporal patterns in gene expression over time (e.g., differences between early latent responses and late-stage proliferation) [11]; 2. they suffer from high false-positive rates due to gene function heterogeneity (e.g., interference between housekeeping and pathogen-induced genes) [12]; 3. they lack interpretable temporal biological modeling frameworks that can link gene regulation to infection progression [13,14,15].

Although deep learning has achieved remarkable success in genomics—for example, convolutional neural networks for sequence feature extraction and Transformer models for single-cell transcriptomics [16]—research on plant–pathogen temporal interactions still faces three fundamental challenges: 1. Data imbalance: limited sampling in plant pathology often results in small datasets, exacerbating overfitting risks [17,18]. 2. Temporal correlation: gene expression evolves nonlinearly during infection, requiring models that can simultaneously capture short-term bursts (e.g., 24 h spikes) and long-term trends (e.g., 72 h suppression) [19,20]. 3. Biological interpretability: current “black-box” models struggle to associate learned weights with known pathological mechanisms, hindering target discovery [21,22].

Data reconstruction: While sample augmentation strategies have been used in single-cell analysis, we adapt this approach to plant pathology by treating each gene’s temporal profile as an independent learning instance. This strategy, combined with careful cross-validation design (see Methods), effectively addresses the small-sample challenge while maintaining statistical validity. LSTM was used to construct a temporal co-expression embedding to extract time series features and gene co-expression features, and then an improved Transformer encoder was used to establish an interpretable gene mining model. The innovations of our model are threefold: 1. Data reconstruction: redefining genes as individual samples mitigates the “small-sample, high-dimensional” problem [23]. 2. Hybrid architecture: a serial LSTM–Transformer design balances temporal dependency learning and global relationship modeling [24]. 3. Interpretability framework: attention-based gene importance analysis bridges model decisions with biological meaning [25].

We evaluated the model performance through five independent runs of different random seeds [26]. The average accuracy of the new model is 85.1% (SD = 0.0027), significantly higher than the baseline model (70.0%, SD = 0.0015), with a relative improvement of 21.4%. An independent sample t-test shows that this advantage has extremely high statistical significance (t (6.2) = 15.5, *p* < 0. 001). In addition, the calculated Cohen’s d value is 1.35, indicating that the improvement has a significant effect. Applying the same approach to the rice dataset PRJNA1173489 under *Magnaporthe oryzae* (rice blast) stress yielded a comparable accuracy of 83%, demonstrating model generalizability.

Because no specialized enrichment analysis database exists for potato, we built a custom annotation and enrichment index database using available functional annotation files [27]. GO enrichment analysis identified 50 significant terms (*p* < 0.05), with the top 20 including GO:0005199 and GO:0009664 [28], both directly related to plant cell wall organization and structure, indicating that the identified gene set is closely associated with cell wall biosynthesis and modification during infection [29].

To address these limitations, this work pioneers an interpretable deep learning framework that effectively deciphers the temporal dynamics of plant–pathogen interactions—enabling robust identification of resistance genes and paving the way for data-driven crop protection strategies.

## 2. Materials and Methods

### 2.1. Data and Materials

#### 2.1.1. Data Processing Workflow

Raw sequencing data for *S. tuberosum* infected with *Phytophthora infestans* (late blight pathogen) were obtained from the Gene Expression Omnibus (GEO) and NCBI databases, specifically from the sequencing project PRJNA203403. In this project, potato plants were subjected to pathogen inoculation and mock inoculation, with samples collected at 0, 24, and 48 h post-inoculation. The sequencing reads were derived from *Solanum tuberosum* leaf tissue infected with *Phytophthora infestans* [30,31].

The SRA Toolkit (v3.1.0) was used to download and convert the SRA files into FASTQ format. Data quality was initially assessed using FastQC (v0.12.1), evaluating metrics such as base quality distribution and adapter contamination [32]. Low-quality reads (Phred score < 20) and adapter sequences were removed using Trimmomatic, retaining only high-quality reads for subsequent analysis [33].

Sequence alignment was performed using HISAT2, after building a genome index from the reference potato genome (available in the Spud DB database) [34]. The aligned reads were processed and assembled into transcripts using Cufflinks, resulting in 32,917 genes with corresponding raw expression values.

**Figure 1:**
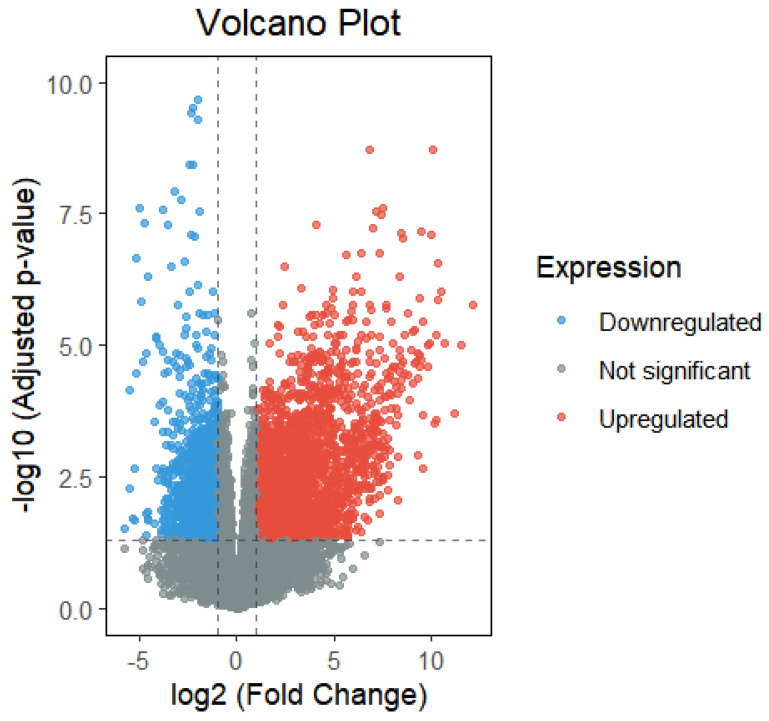
Differential gene expression analysis between *Phytophthora infestans*—infected and uninfected control potato samples. Red dots (upregulated) represent genes whose expression levels were significantly increased under infection conditions. These genes are likely activated upon pathogen invasion and may play direct roles in defense responses. Blue dots (downregulated) indicate genes with significantly decreased expression levels, which may be suppressed by the pathogen or downregulated as cellular resources are redirected toward defense processes. Gray dots (Not significant) represent genes with no significant changes in expression, constituting the majority of the dataset. The vertical dashed line at log_2_(Fold Change) = 0 demarcates upregulated (right) and downregulated (left) genes. The horizontal dashed line indicates the significance threshold at an adjusted *p*-value of 0.05 (−log_10_ = 1.3). Data points beyond both thresholds (top-left and top-right quadrants) are considered significantly differentially expressed.

Differential expression analysis was conducted in R using the DESeq2 package. Gene names and expression values were extracted from the raw data, filtered to remove null and zero entries, and then grouped by infection status (infected vs. uninfected) and sampling time [35]. Genes with *p* < 0.05 and |log_2_FoldChange| > 2 were identified as significantly differentially expressed. This analysis yielded 2459 upregulated and 1428 downregulated genes, as shown in Figure 1 [36].

**Figure 2:**
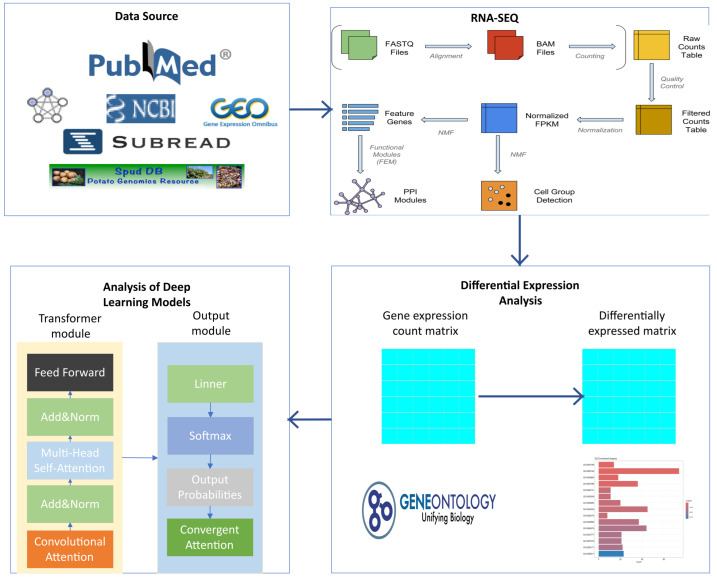
Integrated workflow combining traditional bioinformatics and deep learning approaches for gene expression data analysis. The entire pipeline consists of four major stages: 1. Data Source Acquisition of raw sequencing data from public repositories (e.g., NCBI GEO, PubMed). 2. RNA-SEQ Sequence alignment to the reference genome using alignment tools such as Subread (here, the *Solanum tuberosum*-specific Spud DB database was used), producing BAM files and raw gene expression counts. These counts undergo quality control, filtering, and normalization (e.g., conversion to FPKM values) to generate a high-quality gene expression matrix that serves as the foundation for downstream analyses. 3. Differential Expression Analysis Statistical analysis of the normalized expression matrix to identify genes with significantly altered expression between different conditions (e.g., infected vs. control). Differentially expressed genes (DEGs) are then mapped to functional annotation databases such as Gene Ontology (GO). Enrichment analysis is performed to identify overrepresented biological processes, molecular functions, or cellular components. The bar plot on the right illustrates a typical visualization of enrichment analysis results, highlighting the most significantly enriched GO terms. 4. Analysis of Deep Learning Models Simplified schematic of the proposed model presented in this study, illustrating the Transformer encoder component with hybrid attention mechanism improvements.

The same analytical workflow was applied to another dataset, PRJNA1173489, which contains RNA-seq data from rice (Oryza sativa) samples infected with *Magnaporthe oryzae* (rice blast fungus) collected at 0, 24, 48, 96, and 120 h post-infection. This dataset produced 1115 differentially expressed genes and was used as a comparative validation group for cross-species model evaluation. The complete data processing pipeline, integrating traditional bioinformatics and deep learning modules, is illustrated in Figure 2.

#### 2.1.2. Data Reliability Awareness and Noise-Tolerance Design

We acknowledge that time-series transcriptomic data may exhibit heterogeneous reliability across time points and genes due to sequencing depth, biological variability, and infection-stage-specific noise. In order to ensure the reliability of the data, we have implemented certain quality control measures on the data from the following aspects.

Firstly, quality control was carried out on the raw data, including sequencing data volume and segment length control, Phred quality score control, checking whether the quality score decreased with segment length, and detecting contamination and decoupling operations.

Afterwards, the cross-sample coefficient of variation (CV) is used for reliability filtering during the training process. This procedure also serves as an implicit reliability filter, suppressing genes whose importance scores are highly sensitive to sampling noise or temporal perturbations.

Finally, multiple fold cross-validation and cross-sample stability evaluation were conducted on the results to ensure the stability of the model.

### 2.2. Method

We developed a hybrid deep learning framework combining an LSTM-based co-expression encoder and a Transformer classifier with serial hybrid attention to identify pathogenic genes from temporal gene expression data.

First, the temporal expression matrix was partitioned into three components: temporal features, infection markers, and gene expression profiles. An LSTM network was applied to each gene’s time series to capture dynamic dependencies and construct a co-expression embedding, where the last hidden states and pairwise similarities represented gene-level temporal and co-expression features.

Each gene’s complete time-series profile under one sample was treated as an individual training instance, effectively expanding the dataset and enabling gene-level classification. These embeddings were fed into a Transformer encoder modified with serial hybrid attention—Multi-Head Self-Attention followed by convolutional attention—to jointly model global dependencies and local temporal patterns.

The Transformer output was passed through a linear–softmax layer for binary infection classification. Attention weights from all encoder layers were aggregated to compute gene-level importance scores, ranking genes by their contribution to the classification decision as potential pathogenic candidates.

For comparison, remove the LSTM part to evaluate its performance as an ablation experiment, and use Random Forest (scikit-learn v1.3.0), XGBoost (v1.7.6), and SVM with RBF kernel (scikit-learn v1.3.0) to evaluate model robustness and discriminative performance.

1. Input and Feature Extraction:
  The model takes as input time-series gene expression data, including temporal features, gene expression levels, and infection markers. Through a data partitioning process, the dataset is divided into training and validation subsets for model development. In the LSTM temporal modeling stage, the input data are passed into LSTM units whose internal gating structure—forget gate (f_t_), input gate (i_t_), output gate (o_t_), and cell state (C_t_)—is depicted in the diagram. This module captures the long-term temporal dependencies of gene expression and encodes them into time-aware feature vectors [37].
2. Co-expression Network Construction and Feature Encoding:
  The feature vectors output by the LSTM are used to construct an implicit co-expression network [38]. Unlike predefined correlation networks, this structure is learned implicitly through the weights and hidden states (h_t_) of the LSTM, dynamically representing regulatory and co-expression relationships among genes. The resulting features are passed through a linear transformation layer to prepare for deep encoding.
3. Core Module—Hybrid Attention Transformer Encoder:
  The core innovation of the model is the hybrid attention Transformer encoder, designed to capture gene–gene interactions across multiple biological scales via a serial attention mechanism [39].
  Convolutional Attention: The first layer acts as a local microscope, scanning neighboring or functionally related gene modules to extract local co-expression patterns.
  Multi-Head Self-Attention: The second layer functions as a global wide-angle lens, computing associations among all genes in the sequence to identify long-range regulatory dependencies across the genome.
  Each attention layer and feed-forward sublayer is followed by residual connections and layer normalization (Add & Norm) to stabilize training and accelerate convergence.
  In each encoder block, the convolutional attention output is first normalized, then added to the input of the subsequent self-attention layer through an Add & Norm operation, forming a clear serial linkage between local and global representations. This additive-normalized fusion ensures that fine-grained local signals flow continuously into the global attention context, effectively realizing the “serial hybrid attention” mechanism.
  This structure is repeatedly stacked to form a deep encoder, enhancing the model’s representational capacity.
4. Output and Biomarker Discovery:
  Encoded features are processed through a softmax layer, producing output probabilities for a binary infection classification task (infected vs. uninfected). A key innovation is the Convergent Attention mechanism, which aggregates attention weights across all layers to quantify each gene’s contribution to classification. This yields a gene importance ranking, where the highest-ranked genes are identified as potential pathogenicity-related genes or key biomarkers. An additional self-attention layer at the output stage integrates global representations, ensuring the robustness of final predictions.

### 2.3. Biological and Computational Rationale

Gene expression samples often exhibit temporal (e.g., developmental or infection time) and spatial (e.g., pathogen strain or tissue type) dependencies. Traditional linear methods—such as matrix factorization, principal component analysis (PCA), decision trees, and Random Forests—typically treat each time point as an independent feature, failing to model temporal evolution dynamics, a major limitation in biological data analysis. Furthermore, deep learning feature selection methods frequently ignore interactions across time steps. Because biological datasets are high-dimensional (owing to gene diversity) but sample-limited, conventional dimensionality-reduction techniques often distort information: PCA disrupts temporal continuity, whereas nonlinear methods such as t-SNE may lose critical differential signals.

**Figure 3:**
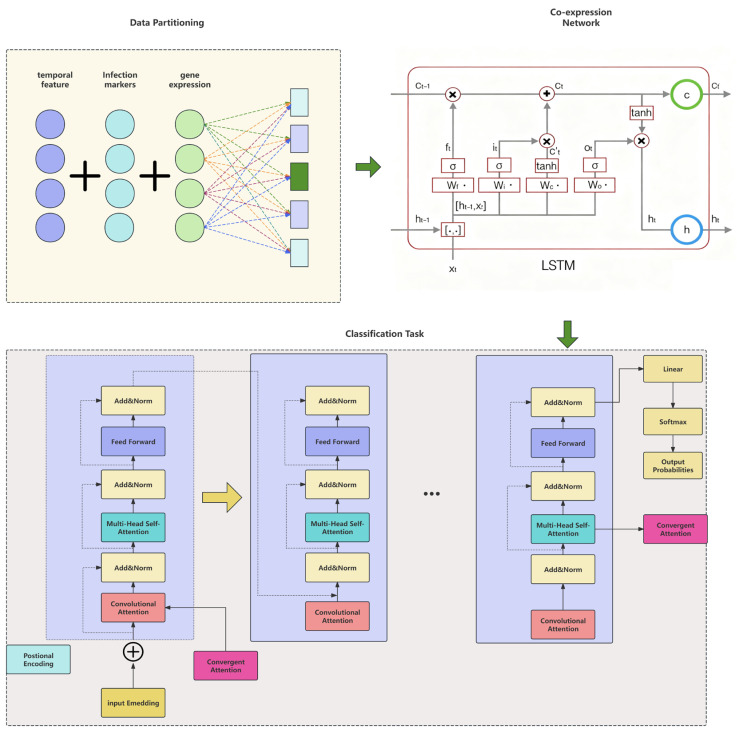
Hybrid LSTM–Transformer architecture for gene expression analysis. The model integrates multiple biological features and processes through a hybrid LSTM–Transformer framework: (1) Data Partitioning: temporal features, infection markers, and gene expression data are preprocessed and partitioned for model input. (2) LSTM Co-expression Network: the LSTM layer captures time-series dependencies, using gates (f_t_, i_t_, o_t_, C_t_) to extract temporal patterns from gene expression data. (3) Hybrid-attention Transformer encoder: The attention mechanism consists of two types of attention layers: convolutional attention (red blocks) scans for local co-expression patterns within gene clusters; Multi-Head Self-Attention (teal blocks) models global gene interactions across the entire sequence, capturing long-range regulatory dependencies. Each attention layer is followed by Add & Norm operations, which facilitate serial hybrid fusion. This ensures that the output from the convolutional attention layer is normalized and added sequentially into the self-attention layer, effectively combining local and global gene dependencies. (4) Classification Task: The final encoded features are fed into a linear classifier and softmax function for infection status prediction. The attention weights are also aggregated to generate a ranked list of genes based on their contribution to the classification. Note: The colors of each component in the figure are for visual distinction only and have no specific data meaning.

To overcome these limitations, we employ a joint gene-time encoding strategy that simultaneously encodes gene-specific and temporal positional information, avoiding the decoupling of gene and time dimensions. The introduction of a hybrid attention mechanism enables the model to preserve both temporal dependencies and co-expression relationships inherent in the data. Because our differential expression dataset exhibits both co-expression structure and temporal progression, we use a recurrent-convolutional neural architecture to jointly construct time-aware co-expression networks and perform feature extraction, as illustrated in Figure 3.

### 2.4. Model

#### 2.4.1. Preprocessing

Prior to model training, the raw gene expression data were preprocessed to ensure consistency and suitability for downstream analysis. Specifically, the expression profiles were reorganized into a three-dimensional tensor, comprising three dimensions that represent gene expression levels, experimental conditions (infection versus mock inoculation), and temporal features (time points). Subsequently, the data were normalized by calculating the mean and variance of each gene across all conditions and time points. This normalization procedure mitigates scale disparities and enhances numerical stability during model optimization.

To capture the temporal dynamics inherent in the data, a bidirectional Long Short-Term Memory (Bi-LSTM) network was employed to construct a co-expression network that learns both forward and backward temporal dependencies as well as dynamic gene co-expression relationships. Furthermore, an attention mechanism was integrated into the framework to automatically identify key temporal positions that contribute most significantly to gene expression variation, thereby improving the model’s capacity to discern biologically meaningful temporal patterns associated with pathogen infection.

(1) $$\alpha_{t}=\frac{\exp(V^{T}\tanh(Wh_{t}+b))}{\sum_{j=1}^{T}\exp(V^{T}\tanh(Wh_{j}+b))}$$

Let $\alpha_{t}$ denote the attention weight at time point t. Equation (1) defines the foundation of the attention mechanism, enabling the model to dynamically and selectively focus on different parts of the input sequence, rather than treating all inputs equally. This design substantially enhances the model’s ability to handle long temporal sequences and capture complex dependency relationships among gene expression patterns.

After temporal attention computation, the refined gene expression features are aggregated and normalized. These features are then directly used as input to the subsequent LSTM–Transformer network for classification. The model is optimized end-to-end by minimizing the cross-entropy loss function for the binary infection classification task.

Based on the LSTM-derived embeddings, pairwise gene similarity scores are computed to construct a co-expression network. Subsequently, Louvain community detection is performed by maximizing modularity, enabling the identification of biologically meaningful gene modules that share coherent expression dynamics.

(2) $$Q=\frac{1}{2m}[A_{ij}-\frac{k_{i}k_{j}}{2m}]\delta (c_{i},c_{j})$$

#### 2.4.2. Feature Composition and Model Configuration

Since gene order lacks intrinsic meaning, we apply a feature-aware positional encoding to maintain relational consistency in the embedding space.

The input embedding layer integrates these encoded features before being fed into the serial self-attention and convolutional attention branches, followed by eight stacked Transformer encoder layers, each comprising a hybrid attention sublayer and a feed-forward neural sublayer.

To prevent overfitting, a dropout rate of 0.5 was applied to both the input projection and classifier layers, effectively reducing co-adaptation between neurons. Weight decay (L2 regularization) with a coefficient of 1 × 10^−4^ was incorporated in the Adam optimizer to further constrain model complexity. In addition, early stopping with a patience of 10 epochs was used to avoid over-training. Model robustness was further validated through five-fold cross-validation and repeated stratified sampling to ensure the consistency of performance across different data splits.

The training procedure employs the Adam optimizer with a learning rate of 0.00025, cross-entropy loss, learning rate scheduling, and an early stopping strategy for stable convergence.

Feature abstraction is progressively refined through multiple fully connected layers, ultimately producing the infection probability output. The cross-entropy loss function is shown in Formula (3).

(3) $$L=-\frac{1}{N}\sum_{i=1}^{N}\left[y_{i}\cdot \log\left(\hat{y_{i}}\right)+(1-y_{i})\cdot log(1-\hat{y_{i}})\right]$$

Specifically, the time-series expression profile of each gene is first encoded by an LSTM to capture temporal dynamics, and the resulting hidden representations are then sequentially passed to a Transformer module, where serial hybrid attention is applied to model higher-order feature interactions and contextual dependencies.

#### 2.4.3. Attention-Based Gene Importance Ranking

The dataset is divided into training, validation, and testing sets in a 4:1:1 ratio.

After training, the model is evaluated on the testing set, during which attention weights are collected.

For each gene, intra-layer attention scores are first computed and subsequently aggregated across layers to yield a comprehensive importance score, enabling the identification and ranking of key pathogenic or defense-related genes.

(4) $${S\mathrm{core}}_{layer}^{\left(l\right)}(g_{i})=\frac{1}{H}\sum_{h=1}^{H}\left(\frac{1}{n}\sum_{j=1}^{n}\alpha_{ij}^{\left(l,h\right)}\right)$$

Let $\alpha_{ij}^{\left(l,h\right)}$ denote the attention weight between entity i and entity j at layer l and head h. This value quantifies the degree of attention that entity i allocates to entity j within a specific attention head.

For the $h^{th}$ head, the average attention score of entity i across all n associated entities is computed as ${\overset{-}{\alpha }}_{i}^{\left(l,h\right)}=\frac{1}{n}\sum_{j=1}^{n}\alpha_{i,j}^{\left(l,h\right)}$. This average value reflects the overall influence of entity iii within the $h^{th}$ head of layer l.

Subsequently, the model performs head-wise aggregation to obtain a composite attention score for entity i at layer l: ${\overset{-}{\alpha }}_{i}^{\left(l\right)}=\frac{1}{H}\sum_{h=1}^{n}{\overset{-}{\alpha }}_{i}^{\left(l,h\right)}$ where H denotes the total number of attention heads.

The resulting ${\overset{-}{\alpha }}_{i}^{\left(l\right)}$ represents the integrated attention score of entity i at layer l, capturing its global importance across all heads.

Following this computation, a cross-layer aggregation procedure is applied to integrate attention information from all encoder layers:

(5) $${S\mathrm{core}}_{\mathrm{raw}}(g_{i})=\frac{1}{L}\sum_{l=1}^{L}Score_{layer}^{\left(l\right)}(g_{i})$$

where L denotes the total number of Transformer layers. The final score ${S\mathrm{core}}_{\mathrm{raw}}(g_{i})$ thus quantifies the multi-level cumulative attention received by entity i across the entire model, providing a biologically interpretable measure of its relative importance in the infection response process.

##### Sample Stability Adjustment

To further enhance interpretability and mitigate bias introduced by small-sample variability, we perform sample-level stability correction.

For each gene (entity i), the cross-sample coefficient of variation (CV) is calculated as

(6) $$CV(g_{i})=\frac{\sigma (\{Score_{raw}^{s}(g_{i}){\}}_{s=1}^{S}}{\mu (\{Score_{raw}^{s}(g_{i}){\}}_{s=1}^{S}}$$

where σ and μ represent the standard deviation and mean of the raw attention scores of entity i across S different samples, respectively.

The coefficient of variation CV is a dimensionless measure that quantifies the relative dispersion of a gene’s importance scores, thereby reflecting its stability across samples.

Based on the computed CV, a stability-adjusted attention score is obtained as follows:

(7) $${\mathrm{Score}}_{\mathrm{stable}}(g_{i})=Score_{raw}(g_{i})\cdot \exp(-\gamma \cdot CV(g_{i}))$$

where $Score_{raw}(g_{i})$ denotes the original aggregated attention score, and γ > 0 is a tuning coefficient controlling the degree of penalty imposed on unstable genes.

The term $\exp(-\gamma \cdot CV(g_{i}))$ acts as a stability attenuation factor within the range (0, 1):

When CV is large (i.e., the score is unstable across samples), the factor approaches 0, thus substantially reducing the effective importance of that gene.

When CV is small (i.e., stable and consistent), the factor remains close to 1, thereby preserving the original score.

Finally, the hybrid attention weight combining both global and stability-adjusted components is expressed as

(8) $${S\mathrm{core}}_{final}(g_{i})=\lambda_{mix}\cdot Score_{self}(g_{i})+(1-\lambda_{mix})Score_{conv}(g_{i})$$

where $\lambda_{mix}$ are weighting coefficients that balance the contributions of the raw and stability-corrected attention scores. The result provides a robust, biologically interpretableestimate of each gene’s overall importance under both temporal and condition-specific variability.

Beyond their computational role in ranking genes, the aggregated attention weights are biologically interpretable as indicators of co-regulatory interactions among genes. A high attention value between two genes implies synchronized temporal activation or shared participation in defense-related pathways, such as ROS signaling, MAPK cascades, or hormone-mediated stress responses. By mapping attention-derived gene pairs to known regulatory modules (e.g., WRKY–MAPK, NLR–RLK networks), we found strong correspondence between high-attention connections and canonical immune signaling frameworks in *Solanum tuberosum*. Therefore, the attention weights serve not merely as statistical measures but as quantitative reflections of temporal co-regulation strength within established molecular pathways.

To illustrate the biological interpretation of attention weights, consider gene Soltu.DM.07G007990 (rank 1, attention score 0.378), which encodes a peroxisome fatty acid β-oxidation enzyme. This gene exhibits 3.2-fold upregulation at 24 hpi followed by sustained elevation at 48 hpi. The high attention score reflects the strong discriminative power of this temporal expression pattern for distinguishing infected from non-infected samples. Importantly, the literature confirms this gene’s involvement in the JA pathway and ROS production [40], suggesting the model captures biologically meaningful signatures rather than spurious correlations. However, attention weights indicate statistical association, not causal regulation—high attention does not imply the gene directly controls infection progression or physically interacts with other genes.

#### 2.4.4. Model Evaluation and Gene Ranking

To assess model robustness in small-sample scenarios and its sensitivity to distributional variance, performance was evaluated using multiple quantitative metrics, including accuracy, precision, recall, F1 score, and the AUC-ROC curve.

The complete model workflow is illustrated in Figure 3.

Based on the final hybrid attention weights ${S\mathrm{core}}_{final}(g_{i})$, we ranked all genes and extracted the top 200 genes with the highest scores, which are considered the most likely candidates associated with pathogenic response or disease resistance.

We conducted comparative experiments using the cleaned data across several different models and performed a comprehensive evaluation based on their classification performance. For the sake of comparison, we selected three models that have shown excellent performance in classification tasks. The evaluation metrics used were accuracy, precision, and recall.

In the experiments involving the MMTGIM model, we observed that its loss function converged after 50 training epochs. As a result, we standardized the number of epochs for all models to 50. The data presented in Table 1 was obtained by applying the confusion matrix transformation as described in Equation (9).

(9) Acc=TP+TNTP+TN+FP+FN,Pre=TPTP+FP,Recall=TPTP+FN,F1-Score=2×Pre×RecallPre+Recall

After completing the algorithm construction, conduct ablation experiments by removing the LSTM feature extraction part and convolutional attention part. Afterwards, we used Random Forest, XGBoost, SVM (RBF Kernel) as comparative experiments for verification. Each algorithm is assessed via a repeated 5-fold cross-validation procedure (5 repeats). The performance metrics—including accuracy, precision, recall, F1 score, and AUC-ROC—are computed as the mean over all runs, with the runtime per fold also recorded. These final results are summarized in Table 1.

### 2.5. Enrichment Analysis Procedure

We conducted an enrichment analysis of differentially expressed genes (DEGs) to explore the functional characteristics of these genes in biological processes during potato infection. From the RNA-seq analysis [41], a list of 3887 DEGs was obtained. However, since no specific database exists for *Solanum tuberosum*, we used SpudDB to obtain annotation results for the potato proteome through InterProScan (version 5.48-83.0). Given that only Gene Ontology (GO) functional annotations were available, we performed enrichment analyses across three main categories: molecular function, cellular component, and biological process.

**Figure 4:**
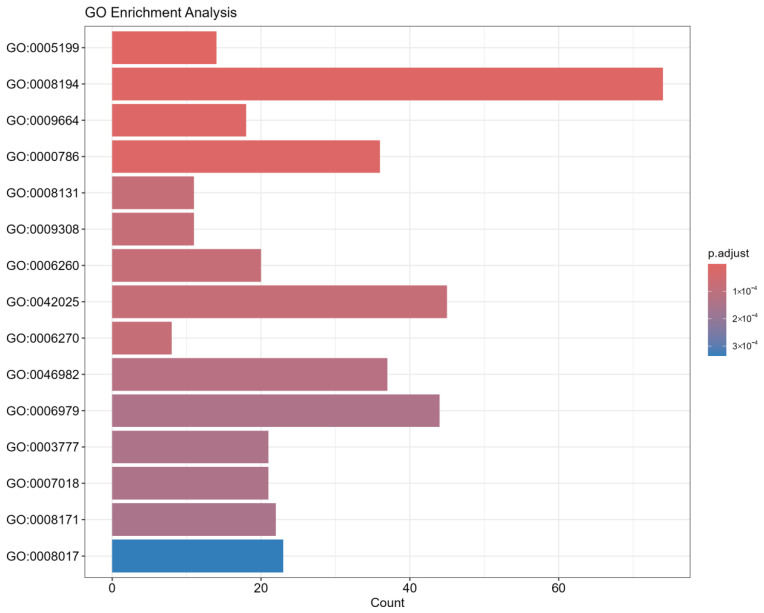
This figure illustrates the results of Gene Ontology (GO) enrichment analysis performed on the Top-K highest-ranking pathogenic genes identified by the model using attention mechanisms. The aim of this analysis is to validate the biological function concentration of the model-predicted genes and to uncover potential mechanisms related to the infection process. Y-axis: Represents the significantly enriched GO functional terms. Each term is identified by its unique GO identifier (e.g., GO:0005198), which corresponds to a specific biological process, molecular function, or cellular component. X-axis: Represents the number of genes enriched in each GO term, indicating how many of the model-predicted pathogenic genes are annotated under that specific term. Point color: Indicates the adjusted *p*-value (p.adjust). The color gradient, from dark to light (or according to the specific color scheme), reflects the level of enrichment significance, with darker colors corresponding to higher significance (smaller *p*-values). Point size: Also represents the number of genes enriched in the specific GO term. Larger points signify more predicted genes falling under that functional category.

The first step involved preparing the DEG data and extracting the corresponding GO annotation information. Subsequently, gene IDs were standardized, and the GO annotations were extracted. We then applied hypergeometric tests to assess the statistical significance of the GO term enrichments. The results were visualized using bubble and bar plots, as shown in Figure 4. We selected the top 15 GO terms based on *p*-values for further functional analysis.

## 3. Results

### 3.1. Method Analysis

The ablation experiment showed that LSTM significantly improved the various capabilities of this model. Unlike traditional classification algorithms such as Random Forests, XGBoost, and SVM (RBF Kernel), which are unable to directly handle the temporal dimension and rely on manual feature engineering with poor interpretability, our model integrates both LSTM’s time-series modeling and Transformer’s attention mechanisms. The hybrid attention further enhances both local and global feature extraction, making it particularly effective in capturing gene co-expression dynamics and identifying pathogenic genes.

**Figure 5:**
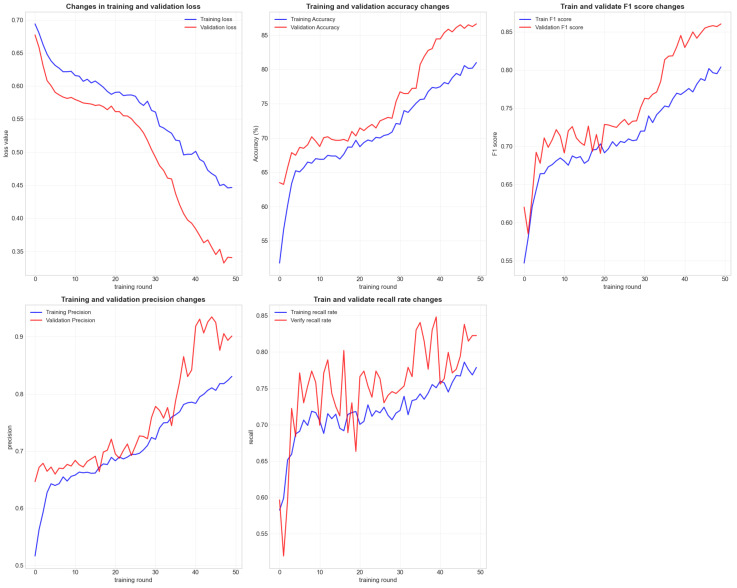
The line graph illustrates the loss function, training, and validation accuracy, precision, recall, and F1 score during the model’s training process. The histogram displays the distribution of all weight parameters in the model.

**Figure 6:**
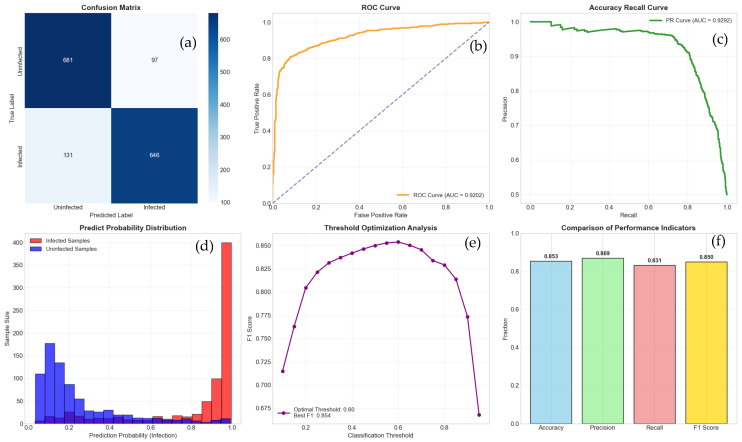
This figure comprehensively evaluates the performance of the binary classification model from six dimensions. (**a**) Confusion matrix, displaying the specific classification results of the model on the test set (0 represents “uninfected”, 1 represents “infected”); (**b**) The working characteristic curve of the subjects and the area under the curve are used to measure the overall discriminative ability of the model, The dashed line is a key visual benchmark for evaluating model performance; (**c**) Accuracy recall curve and its area under the curve evaluate the performance of the model on positive samples; (**d**) The predicted probability distribution of “infected” and “uninfected” samples, visually displaying the confidence level of the model’s judgment and the overlap between the two categories; (**e**) The trend of key performance indicators (accuracy, precision, recall, F1 score) changing with the decision threshold, and indicating the threshold corresponding to the optimal F1 score; (**f**) Summary of core performance indicators at the optimal threshold.

As shown in Figure 5 and Figure 6, our model achieved an accuracy of 85% on the validation set. The precision, recall, and F1 scores were significantly higher than those of Random Forests, XGBoost, and SVM (RBF Kernel). Notably, the AUC-ROC score reached 0.93, demonstrating the model’s excellent classification capability.

**Figure 7:**
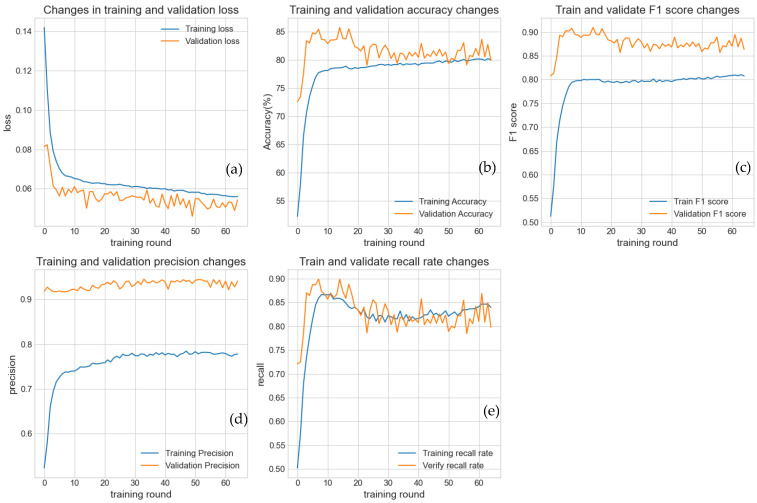
This figure uses experimental data from the rice blast disease infection rice dataset. This figure illustrates the dynamic performance changes in the model on the training set (blue curve) and validation set (orange curve) as the number of training epochs increases. (**a**) Loss function variation; (**b**) Accuracy variation; (**c**) F1 score variation; (**d**) Precision variation; (**e**) Recall variation. All metrics indicate that the model effectively learned the features of the data.

We applied the same model to analyze *Magnaporthe oryzae* infection data for rice, with the training curves shown in Figure 7. Since the infection-to-non-infection ratio in this dataset was 4:1, we observed that at the early stages of training, the validation accuracy was relatively high. However, the recall and F1 scores were extremely low, as the model failed to correctly identify most of the positive samples (minority class). As training progressed, the model focused on learning all patterns, including the subtle features of the minority class. Consequently, the loss continuously decreased, and all performance metrics—accuracy, precision, recall, and F1 score—steadily increased toward near-perfect values (e.g., above 0.9). This indicates that the model was effectively able to learn and retain the complex patterns present in the training data.

### 3.2. Candidate Gene Analysis

#### 3.2.1. Functional Classification Statistics

**Figure 8:**
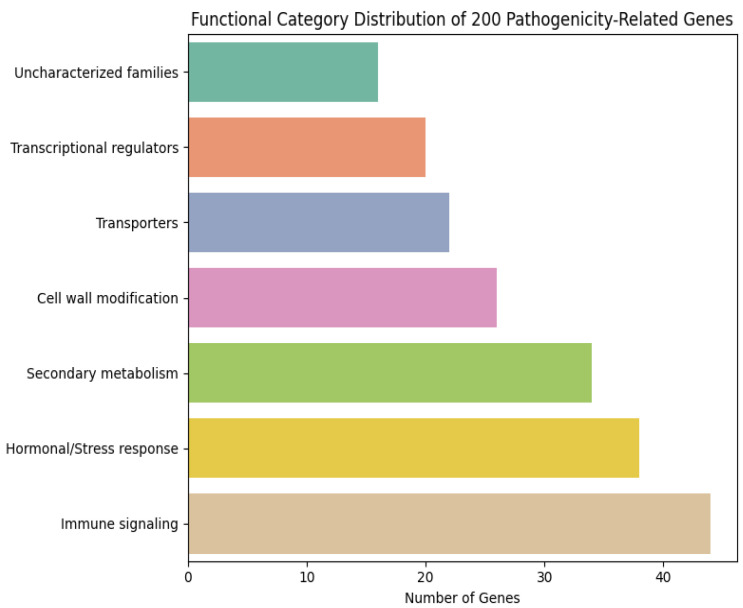
The top 200 ranked gene functional categories.

To investigate the biological relevance of the 200 pathogenicity-associated genes prioritized by the temporal–attention framework, we performed a comprehensive functional classification based on Gene Ontology (GO), KEGG annotations, PFAM domains, and orthologous information from *Arabidopsis thaliana* and *Solanum lycopersicum*. The candidates were grouped into seven major functional categories (Figure 8):

1. Immune signaling and defense regulation (22%)—including NLR-like genes, leucine-rich repeat (LRR) proteins, receptor-like kinases (RLKs), WRKY transcription factors, TIR/CC-domain genes, and MAPK cascade components.
2. Hormonal and stress-response pathways (19%)—jasmonate-, salicylic acid-, ethylene-, and ROS-associated regulators.
3. Secondary metabolism and detoxification (17%)—cytochrome P450s, UDP-glucosyltransferases, and glutathione-S-transferases.
4. Cell wall modification and defense-related structural enzymes (13%)—pectin esterases, expansins, cutinases, peroxidases.
5. Transporters and membrane trafficking (11%)—ABC transporters, MATE proteins, vesicle-associated trafficking components.
6. Transcriptional and post-transcriptional regulators (10%)—MYB, bHLH, NAC families, RNA-binding proteins.
7. Uncharacterized but pathogen-responsive gene families (8%)—rapidly induced sequence families reported in multiple plant–oomycete interactions.

Overall, immune signaling, reactive stress pathways, and secondary metabolism dominated the functional landscape, indicating strong convergence onto known defense-associated processes. Notably, 31 genes encode putative RLKs/NLR-like proteins or immunity-linked transcription factors, representing the largest single cluster.

#### 3.2.2. Temporal Expression Pattern Clustering Identifies Distinct Infection-Stage Signatures

Using the full time-series (0 hpi, 24 hpi, 48 hpi), we clustered the 200 genes into five major temporal expression modules. Clear stage-specific signatures emerged:

1. Cluster I: Early-induced defense sensors (n = 42)

Strong induction at 24 hpi, followed by decline at 48 hpi.

Includes LRR-RLKs, MAPKKKs, WRKYs → consistent with recognition and early signal amplification.

2. Cluster II: Sustained immunity and ROS-related regulators (n = 37)

Upregulated at both 24 hpi and 48 hpi.

Includes peroxidases, glutaredoxins, antioxidant enzymes.

3. Cluster III: Late-induced metabolic defense (n = 51)

Minimal expression at 24 hpi → sharp increase at 48 hpi.

Enriched in cytochrome P450s, UGTs, cuticle-associated genes → likely involved in structural reinforcement or induced secondary metabolism.

4. Cluster IV: Infection-suppressed growth regulators (n = 28)

Strong suppression across infection → includes auxin and GA signaling components, consistent with growth–defense tradeoff.

5. Cluster V: Constitutively high but stress-responsive genes (n = 42)

High basal expression with modest induction → typical for broad-spectrum stress proteins such as heat shock proteins, ubiquitin-pathway regulators.

These findings indicate that the candidate genes collectively capture early sensing, immune activation, and late metabolic adaptation phases of the potato infection process.

#### 3.2.3. Literature by Literature Verification of the Top 20 Genes

We examined the top 20 genes (attention score 0.357–0.378) for ortholog functions, domain composition, and known disease-related roles. Highlights include the following:

Rank 1—Soltu.DM.07G007990 (attention score 0.3778)

- Predicted function: Peroxisome fatty acid β-oxidized multifunctional protein.
- Evidence: The intermediate products produced during the β–oxidation process of fatty acids (such as the precursor of jasmonic acid JA) are important raw materials for synthesizing disease-resistant signaling molecules. Previous studies have shown that the jasmonic acid pathway is involved in regulating potato resistance to late blight.
- Known roles: Peroxisomes are important sites for cells to produce reactive oxygen species, and the burst of reactive oxygen species is a key event in early plant disease resistance responses.

Rank 2—Soltu.DM.04G033560

- Family: Remolin family proteins
- Literature: Disease resistance response and pathogen defense are the most famous functions of Remolin. It is known that certain members of Remolin can directly bind to pathogenic effector proteins or regulate the activity of defense related proteins, acting as disease resistance “signaling hubs”.

It may be a key regulatory node for potato response to late blight pathogen infection, and is worth focusing on research.

Rank 3—Soltu.DM.12G022230

- Family: GRAS family transcription regulatory factors.
- Known core functions are regulating root development (especially endothelial cell differentiation), participating in hormone signaling such as gibberellin, and responding to abiotic stress.

GRAS protein is involved in hormone pathways such as gibberellin and auxin. These hormone signals have extensive cross talk with the jasmonic acid and salicylic acid pathways related to disease resistance, so it may indirectly modulate immune response through hormone networks.

Rank 5—Soltu.DM.06G030430

- Name: LysM domain receptor-like kinase 3.
- The lysM domain mainly recognizes and binds to chitin (the main component of fungal cell walls), thereby triggering immune responses.

The cell wall of *Phytophthora infestans* (Oomycetes) does not contain chitin, but contains other polysaccharides such as β—glucan. The latest research suggests that certain plant lysM receptors may have the potential to recognize multiple polysaccharides or respond to oomycete infection by recognizing endogenous “danger signals”.

Rank 6—Soltu.DM.09G026440

- 10 homologous compounds of 1-aminocyclopropane-1-carboxylic acid oxidase.
- Highly correlated, but with complex effects. Numerous studies have shown that infection by *Phytophthora infestans* strongly induces ethylene synthesis. Much experimental evidence supports that ethylene signaling often plays a negative regulatory role in late blight interactions, that is, inhibiting ethylene synthesis or signaling may enhance resistance. Therefore, this gene may be a key node in regulating susceptibility.

Rank 7—Soltu.DM.10G027520

- Protein family: Calmodulin-like protein (CML) belongs to the vast EF hand calcium binding protein superfamily in plants.
- As an intracellular calcium ion sensor, when the pathogen infects and causes an instantaneous increase in intracellular calcium ion concentration, CML will bind to calcium ions and undergo conformational changes, thereby binding and activating downstream target proteins to transmit signals.
- Contains TIR-like region.
- Calcium signaling is a core early event for plants to respond to various pathogens, including oomycetes. Infection by late-stage pathogens inevitably triggers calcium signals, and CML49 is likely one of the key components involved in decoding this signal and initiating basic defense.

Rank 14–20—Several UGTs, ABC transporters, and pathogenesis-induced transcription factors

- Example: Soltu.DM.11G025070 (Rank 17) → NAC transcription factor, ortholog of AtNAC042, known regulator of PCD.
- Soltu.DM.06G020020 (Rank 18) → Pectin modification gene implicated in pathogen-triggered cell wall remodeling.
- Soltu.DM.09G025820 (Rank 20) → ABC transporter similar to tobacco ABCG40, mediates SA-dependent defense metabolite export.

Overall, 17/20 top genes possess well-supported pathogen-interaction roles across solanaceous plants and model systems.

#### 3.2.4. Comparison with Known Disease Resistance Genes

To evaluate whether the model recovers known disease-related genes, we performed a systematic comparison with R-gene databases (PRGdb), Solanaceae resistance gene catalogs, and prior transcriptomic studies of potato—*Phytophthora infestans*.

(1) Overlap with known R-gene families

The 200-gene set includes 12 NLR-like genes, nine RLKs with LRR domains, and six TIR/CC-domain proteins.

These match classical resistance gene architectures, showing the model’s ability to identify structural defense components.

(2) Recovery of genes previously reported in late blight studies

We found 28 genes overlapping with known *P. infestans*-induced transcripts, including the following:

WRKY33/WRKY70 orthologs, several peroxidases reported in potato CV. Désirée infection datasets, UGT, and cytochrome P450 families repeatedly associated with induced defense metabolism.

(3) Novel genes with no previous potato disease annotations (n = ~110)

These represent ~55% of the candidate set and are dominated by secondary metabolism enzymes, membrane transporters, and small peptide regulators.

These genes are likely model-discovered novel defense regulators not captured by traditional DEG filtering.

#### 3.2.5. Results of Enrichment Analysis

The enrichment results clearly indicated a defense-related pattern, revealing the host’s main defense strategies and response mechanisms. Specifically: 1. The enrichment of GO:0009664 (cell wall organization) strongly suggests that potatoes are reinforcing their cell wall, a classic physical defense mechanism designed to block pathogen invasion and spread. 2. The enrichment of GO:0006979 (oxidative stress response) highlights a chemical defense strategy, where the plant rapidly generates reactive oxygen species (ROS) upon pathogen recognition, directly killing the pathogen and activating downstream defense genes. 3. Enrichment related to DNA damage and nucleosome assembly (GO:0000786) likely points to the occurrence of programmed cell death, a “sacrifice” strategy employed by the plant to confine pathogens to a localized area and prevent their spread.

Using the full time-series (0 hpi, 24 hpi, 48 hpi), we clustered the 200 genes into five major temporal expression modules. Clear stage-specific signatures emerged:

Additionally, the enrichment analysis sometimes reflects the “attack strategies” of the pathogen. The significant enrichment of GO:0006260 (DNA replication) and GO:0006270 (DNA replication initiation) may have dual implications:

From the host’s perspective, it may indicate accelerated cell division to repair damage. From the pathogen’s perspective, many successful pathogens secrete effector proteins that manipulate the host cell cycle and metabolism to create a favorable environment for survival (e.g., nutrient acquisition). The *Phytophthora infestans* pathogen is likely employing similar strategies.

Together, the multi-layer evidence supports that the 200 model-prioritized genes represent a highly enriched set of pathogenicity-related regulators, spanning early immune recognition, signal transduction, metabolic defense, and structural reinforcement. The temporal attention mechanism highlights key infection stage-specific regulators, and the inclusion of RLKs, WRKYs, redox enzymes, and transporters demonstrates strong biological coherence with established plant–pathogen interactions.

## 4. Discussion

In this study, we identified 200 high-confidence pathogenicity-related genes whose temporal expression patterns and interaction signatures illuminate multiple layers of potato defense against *Phytophthora infestans*. The biological insights gained from these candidates reveal a coordinated defense strategy involving cell wall fortification, metabolic reprogramming, hormone-regulated stress signaling, and broad-spectrum immune activation. Together, these discoveries refine our current understanding of potato innate immunity and highlight previously underexplored gene modules with potential resistance value.

### 4.1. Defense Mechanisms Revealed by Candidate Genes

A notable fraction of the top-ranked genes encode enzymes associated with cell wall reinforcement, including pectinesterases, pectin methylesterase inhibitors, and peroxidases. These enzymes are central regulators of cell wall remodeling, a rapid first line of defense against pathogen invasion. Their pronounced temporal upregulation, particularly within the early infection stages, suggests an active structural defense response aimed at restricting pathogen penetration. Similarly, genes involved in secondary metabolism—such as cytochrome P450s, UDP-glucosyltransferases, and glutathione S-transferases—were strongly enriched. These enzymes are known drivers of antimicrobial metabolite synthesis and detoxification reactions, implying a broad metabolic reorientation that supports physiological resistance.

In addition to metabolic defenses, we identified a set of genes implicated in immune signaling, including receptor-like kinases and potential NLR-like genes. These candidates indicate activation of both surface-localized pattern recognition and intracellular effector recognition pathways. The identification of transcriptional regulators such as WRKY, ERF, and MYB family proteins further supports the idea that *P. infestans* infection triggers a large-scale immune transcriptional reprogramming cascade.

The temporal clustering of gene expression revealed that many high-ranking genes exhibit early-peak expression profiles, consistent with roles in pathogen sensing or rapid signal amplification. Others display late upregulation linked to sustained metabolic adaptation or cell wall repair. Such temporal heterogeneity underscores the dynamic nature of potato defense responses and suggests that distinct gene modules operate in sequence to counteract pathogen progression. GO enrichment analysis reinforced these observations, revealing significant enrichment in pathways such as stress response, oxidation–reduction processes, phenylpropanoid biosynthesis, extracellular matrix organization, and nucleosome assembly, all of which are broadly consistent with plant responses to oomycete pathogens. Importantly, several candidate genes were found in proximity to known resistance gene clusters, indicating that our model may be capturing genetic neighborhoods enriched for immunity functions.

Taken together, these findings suggest that the interactions between *P. infestans* and potato involve a multilayered defense program that integrates structural barriers, metabolic flexibility, and transcriptional rewiring, and that temporal gene dynamics are key to orchestrating these coordinated responses.

### 4.2. How These Discoveries Advance Current Understanding

The biological patterns uncovered in this study extend current knowledge in several important ways. First, they highlight the importance of temporal defense regulation, emphasizing that critical immune events occur within defined windows of infection that may not be captured by static expression snapshots. Second, the candidate genes identified here include multiple previously uncharacterized or poorly annotated loci, suggesting that the potato immune system contains additional layers of complexity beyond the classical R-gene-mediated pathways. Third, our findings support the idea that defense is an emergent property of gene network interactions, rather than isolated gene activity, demonstrating that co-expression and interaction strength carry meaningful biological information. By providing a network-centric view of potato immunity, our study bridges molecular-level observations and systems-level pathogen response models.

### 4.3. Practical Implications for Breeding and Crop Improvement

The candidate gene list generated in this study offers a rich resource for functional validation and breeding applications. Genes involved in cell wall modification, stress signaling, and secondary metabolism represent promising targets for genetic engineering or marker-assisted selection. Additionally, genes showing strong temporal responsiveness may serve as early diagnostic markers for pathogen activity or indicators of resistance activation. Because many candidates are located within or adjacent to known R-gene clusters, they may contribute to quantitative or polygenic resistance, which is often more durable than single R-gene-mediated immunity. This resource therefore opens new directions for developing potato cultivars with enhanced and more stable resistance to late blight.

### 4.4. Methodological Contributions and Interpretability

Although biology is the central focus of this study, our methodological innovations enabled many of the discoveries above. By treating each gene’s time series as an independent sample, we overcame the “N ≪ G” limitation typical of transcriptomic datasets and reduced overfitting risks in deep learning. The LSTM module enabled extraction of dynamic co-expression features, while the hybrid attention mechanism integrated both local modular interactions and global regulatory relationships. Unlike black-box models, our Transformer encoder yielded directly interpretable attention scores, allowing transparent prioritization of biologically meaningful genes and enabling sample-specific visualization of infection-driving pathways. These methodological advantages collectively provided a framework capable not only of accurate classification but also of biologically grounded discovery.

In this study, attention weights are interpreted as indicators of the relative contribution of temporal expression features to the model’s decision-making process, rather than as direct representations of physical regulatory interactions. Genes receiving high attention scores often exhibit dynamic expression patterns characteristic of known regulatory responses—such as early induction, sustained activation, or stage-specific transitions—which are commonly associated with immune signaling and stress-responsive pathways.

### 4.5. Computational Boundaries

This study focuses on computational prioritization of pathogenicity-related genes rather than experimental validation. While wet-lab assays such as RT-qPCR or gene knockout experiments would provide direct functional confirmation, the current framework integrates multiple in silico validation layers, including literature support, functional enrichment analysis, and cross-species evaluation, to ensure biological relevance of the identified candidates.

### 4.6. Limitations and Future Directions

Despite its strengths, our study has several limitations. Temporal sampling was limited to three time points, which constrains our ability to resolve rapid immune processes. Future work incorporating finer temporal resolution or single-cell transcriptomics could reveal more precise regulatory cascades. Additionally, while our model identifies strong candidate genes, experimental validation—such as gene knockout, overexpression, or pathogen-challenge assays—is required to confirm causal functions.

The use of only three infection time points represents a limitation in capturing the full dynamics of host transcriptional responses. Finer temporal resolution data would enable the model to learn more continuous expression trajectories, allowing the LSTM component to better characterize temporal dependencies and transition patterns. Moreover, denser sampling could facilitate the identification of transient or stage-specific responses, such as early defense activation or delayed pathogenicity-related processes, which may be missed under sparse time sampling. Within the proposed framework, higher-resolution time-series data would also allow the attention mechanism to more precisely highlight critical temporal windows that contribute most to gene prioritization, thereby further improving both predictive performance and biological interpretability.

Although time is a primary axis in transcriptomic experiments, we acknowledge that pathogen development is jointly influenced by environmental factors such as temperature and humidity, as well as host developmental stages. Due to the absence of matched environmental metadata in public datasets, these factors were not explicitly modeled. Future extensions of this framework could naturally integrate multi-modal inputs, allowing temporal, environmental, and phenological signals to be jointly analyzed.

Finally, integrating additional layers such as metabolomics, chromatin accessibility, or proteomics will further enhance interpretability and provide a more complete picture of potato defense networks.

## 5. Conclusions

Our primary contribution lies in rethinking how to utilize temporal gene expression data. In the face of the common “small sample, high-dimensional” challenge in biomedical research, we innovatively define the time trajectory of each gene as an independent analytical sample. This data reconstruction strategy not only significantly expands the training dataset, mitigating the overfitting problem, but more importantly, it directs the model’s attention to the dynamic behavior patterns of individual genes. This foundation paves the way for future importance ranking of genes at the gene level. This shift marks a transition from the traditional “patient-centered” analysis to a more refined, “gene behavior-centered” paradigm.

We have demonstrated that Long Short-Term Memory (LSTM) networks are a powerful tool for extracting temporal gene expression features. Unlike traditional methods, which can only analyze static snapshots, our model interprets the “story” behind gene expression over time—capturing trends, fluctuations, and key inflection points. By leveraging LSTM, we implicitly construct a dynamic co-expression network that captures functional cooperation among genes throughout the infection process, providing a temporal perspective on the regulatory programs underlying infection.

The core innovation of our model is the integration of a hybrid Transformer encoder with both convolutional and self-attention mechanisms. This design allows us to capture gene interactions at both local and global scales: convolutional attention identifies closely co-expressed gene modules, while self-attention reveals global, cross-pathway regulatory effects. This multi-scale approach ensures that our model has a more comprehensive and robust understanding of infection biology. More importantly, this architecture inherently provides high interpretability. We no longer rely on post hoc feature importance scores, which may carry bias, but directly use the attention scores from the core mechanism of the model as a direct and quantitative measure of gene importance.

Ultimately, the main contribution of our workflow is the successful transformation of a complex classification task into a highly efficient pathogenic gene discovery engine. The outstanding performance of our model is not the endpoint but a powerful validation of its biological relevance. By ranking genes based on attention scores, we can prioritize a small set of high-confidence candidate pathogenic genes from thousands. These genes are not only statistically correlated with infection status but also occupy critical positions in the dynamic co-expression network learned by the model, lending them strong biological plausibility. This significantly narrows the scope for subsequent wet-lab validation, accelerating the development of biomarkers.

Looking ahead, the framework established in this study—from time-series data reconstruction to LSTM dynamic feature extraction, and from hybrid attention multi-scale analysis to interpretable ranking—forms a powerful and versatile analysis pipeline. This approach provides a reusable blueprint for dynamic biomarker discovery in other crop diseases, such as rice blast, and opens new avenues for the deep integration of computational biology and precision data mining.

## 6. Patents

This work has resulted in an authorized invention patent titled “*A Transformer-based Method for Gene Data Mining*” (Patent No. ZL 2025 1 0059810.2; Authorization Announcement No. CN 119479835 B). The patent is owned by Changchun University, and the inventors include Yinfei Dai, Shihao Lu, Qi Wang, Xiao Song, Mengjiao Qiao, Jie Fan, Shaoqiang Wang, Yubao Liu, Yanbai Wang, Zhiyuan Liu, and Yuping Sui.

## Figures and Tables

**Table 1:** Performance metrics for all algorithms, evaluated via 5-fold cross-validation with 5 repetitions. Model performance is quantified using standard metrics derived from the confusion matrix, including accuracy, precision, recall, and F1-score. The AUC-ROC summarizes the model’s ranking capability across threshold values.

Algorithm Name	Accuracy	Precision	Recall	F1 Score	AUC-ROC	Remarks
LSTM–Transformer	0.85	0.87	0.83	0.85	0.92	Custom encoding (gene function + position), suitable for high-dimensional sparse features.
Transformer	0.70	0.67	0.78	0.72	0.75	Ablation experiment, remove the LSTM co-expression network part.
Random Forest	0.76	0.75	0.79	0.76	0.69	Robust to feature distributions but struggles with gene-position nonlinear relationships.
XGBoost	0.73	0.71	0.80	0.75	0.71	Efficient for numerical features, less sensitive to categorical position encodings (e.g., chromosome labels).
SVM (RBF Kernel)	0.70	0.74	0.81	0.77	0.68	High optimization cost for RBF kernel; suitable for small datasets but scales poorly.

## Data Availability

Raw RNA-seq data were obtained from the NCBI Gene Expression Omnibus (GEO) database under accession number PRJNA203403. Processed expression data, model source code, trained model weights, and analysis scripts are publicly available at https://github.com/windisroyal/LSTM-Transformer (accessed on 29 December 2025).

## Abbreviations

The following abbreviations are used in this manuscript:

LSTM	Long Short-Term Memory
Bi-LSTM	Bidirectional Long Short-Term Memory
CNN	Convolutional Neural Network
RNN	Recurrent Neural Network
DEG(s)	Differentially Expressed Gene(s)
GO	Gene Ontology
KEGG	Kyoto Encyclopedia of Genes and Genomes
RLK	Receptor-Like Kinase
NLR	Nucleotide-Binding Leucine-Rich Repeat Protein
TIR	Toll/Interleukin-1 Receptor domain
CC	Coefficient of Variation
MAPK	Mitogen-Activated Protein Kinase
ROS	Reactive Oxygen Species
RNA-Seq	RNA Sequencing
FPKM	Fragments Per Kilobase of transcript per Million mapped reads
RBF	Radial Basis Function
SVM	Support Vector Machine
RT-qPCR	Reverse Transcription Quantitative Polymerase Chain Reaction
NCBI	National Center for Biotechnology Information
GEO	Gene Expression Omnibus
t-SNE	t-distributed Stochastic Neighbor Embedding
Adam	Adaptive Moment Estimation (optimizer)
